# Leucine-rich repeat kinase 2 (LRRK2) regulates α-synuclein clearance in microglia

**DOI:** 10.1186/s12868-016-0315-2

**Published:** 2016-11-30

**Authors:** Tatsunori Maekawa, Toshikuni Sasaoka, Sadahiro Azuma, Takafumi Ichikawa, Heather L. Melrose, Matthew J. Farrer, Fumiya Obata

**Affiliations:** 1 Department of Biochemistry, Graduate School of Medical Sciences, Kitasato University, 1-15-1 Kitasato, Minami-ku, Sagamihara, Kanagawa 252-0373 Japan; 2 Department of Laboratory Animal Science, Kitasato University School of Medicine, 1-15-1 Kitasato, Minami-ku, Sagamihara, Kanagawa 252-0373 Japan; 3 Laboratory of Neurochemistry, National Institute for Basic Biology, 38 Nishigonaka Myodaiji, Okazaki, Aichi 444-8585 Japan; 4 Department of Comparative and Experimental Medicine, Brain Research Institute, Niigata University, 1-757 Asahimachidori, Chuo-ku, Niigata, Niigata 951-8585 Japan; 5 Department of Neuroscience, Mayo Clinic, 4500 San Pablo Rd S, Jacksonville, FL 32224 USA; 6 Department of Medical Genetics, Centre for Applied Neurogenetics, Djavad Mowafaghian Centre for Brain Health, University of British Columbia, 2329 West Mall, Vancouver, BC V6T 1Z4 Canada; 7 Division of Clinical Immunology, Graduate School of Medical Sciences, Kitasato University, 1-15-1 Kitasato, Minami-ku, Sagamihara, Kanagawa 252-0373 Japan; 8 R & D Center for Cell Design, Institute for Regenerative Medicine and Cell Design, Kitasato University School of Allied Health Sciences, 1-15-1 Kitasato, Minami-ku, Sagamihara, Kanagawa 252-0373 Japan

**Keywords:** Microglia, LRRK2, α-Synuclein

## Abstract

**Background:**

α-Synuclein (αSYN) has been genetically implicated in familial and sporadic Parkinson’s disease (PD), and is associated with disease susceptibility, progression and pathology. Excess amounts of αSYN are toxic to neurons. In the brain, microglial αSYN clearance is closely related to neuronal survival. Leucine-rich repeat kinase 2 (LRRK2) is the one of the other genes implicated in familial and sporadic PD. While LRRK2 is known to be expressed in microglia, its true function remains to be elucidated. In this study, we investigated αSYN clearance by microglia isolated from LRRK2-knockout (KO) mice.

**Results:**

In LRRK2-KO microglia, αSYN was taken up in larger amounts and cleared from the supernatant more effectively than for microglia isolated from wild-type (WT) mice. This higher clearance ability of LRRK2-KO microglia was thought to be due to an increase of Rab5-positive endosomes, but not Rab7- or Rab11-positive endosomes. Increased engagement between Rab5 and dynamin 1 was also observed in LRRK2-KO microglia.

**Conclusion:**

LRRK2 negatively regulates the clearance of αSYN accompanied by down-regulation of the endocytosis pathway. Our findings reveal a new functional role of LRRK2 in microglia and offer a new insight into the mechanism of PD pathogenesis.

## Background

α-Synuclein (αSYN; *SNCA*) is one of the key molecules involved in familial and sporadic Parkinson’s disease (PD) [1–5]; genomic multiplication and point mutations in the α-*synuclein* gene *(SNCA)* are known to be causal factors for the familial parkinsonism forms of PD, PARK1 and PARK4 [6–9]. αSYN comprises 140 amino acids, which form an amphipathic region, a NAC domain, and an acidic tail [10]. Because of the hydrophobicity of the NAC domain, αSYN easily forms toxic fibrillar structures, and an excess amount of αSYN induces cell death, eventually leading to PD [11–13]. αSYN is expressed in neurons, where it is involved in exocytosis in the presynaptic region [14, 15], and its transmission from cell to cell has been demonstrated [16–18]. These findings suggest that clearance of αSYN in the brain is crucially important for prevention of neuronal cell death.

Microglia are immune cells in the brain playing crucial roles in inflammatory responses, scavenging, and production of neurotropic factors [19]. Accumulated evidence indicates that microglia participate in the pathogenesis of various neurodegenerative diseases [20, 21]. In Alzheimer’s disease, β-amyloid stimulates microglia and induces the production of inflammatory cytokines [22]. Clearance of β-amyloid by microglia has a critical role in prevention of the disease [23, 24]. In PD, microglia are involved in both disease -prevention through clearance of αSYN and disease -progression through production of inflammatory cytokines in which the oligomeric αSYN stimulates toll-like receptor (TLR) 2 whereas a high amount of monomeric αSYN stimulates TLR4 [25–28]. Among the various types of brain cells, microglia are known to have the highest ability to degrade αSYN [29]. One study using mice overexpressing αSYN has shown that acceleration of microglial clearance through αSYN opsonization ameliorated the degree of neurodegeneration [30]. It is believed that achieving adequate degradation of αSYN might be a new therapeutic approach for diseases characterized by pathological accumulation of αSYN [31, 32].

*Leucine*-*rich repeat kinase 2 (LRRK2)* is the gene responsible for autosomal-dominant PD, PARK8, which was originally defined by linkage analysis of a Japanese family (the Sagamihara family) [33–36]. LRRK2 is a complex kinase consisting of LRR, ROC, COR, kinase, and WD40 domains [37]. Accumulated evidence suggests that LRRK2 plays a key role in axonal extension, autophagy, proliferation, and survival of neurons [38, 39]. In addition to neurons, LRRK2 is highly expressed in immune cells such as B cells, macrophages, and microglia [40–43]. Several studies have demonstrated that LRRK2 is related to inflammatory responses of microglia that could be involved in the development and progression of neurodegeneration [44, 45]. It has been reported that pathological mutations of LRRK2 lead to increased production of inflammatory cytokines [46].

With regard to the association between LRRK2 and αSYN, it has been reported that LRRK2 knockout (KO) attenuates the neuropathology that is induced by αSYN overexpression in mouse brain through a delay of neuronal death resulting from improved structure and function of the Golgi complex [47]. Another study has demonstrated that LRRK2-KO ameliorated neurodegeneration in αSYN-overexpressing rats by inhibiting the recruitment of chronically activated proinflammatory myeloid cells [48]. Although these studies suggest an association between LRRK2 and the neuropathology caused by αSYN, no study has yet addressed how microglial LRRK2 is involved in αSYN clearance.

In the present study, we analyzed microglia treated with αSYN and revealed a new functional role of microglial LRRK2. Our results indicate that LRRK2 acts as a negative regulator of αSYN clearance.

## Results

### LRRK2 is a negative regulator of αSYN clearance in microglia

**Fig. 1:**
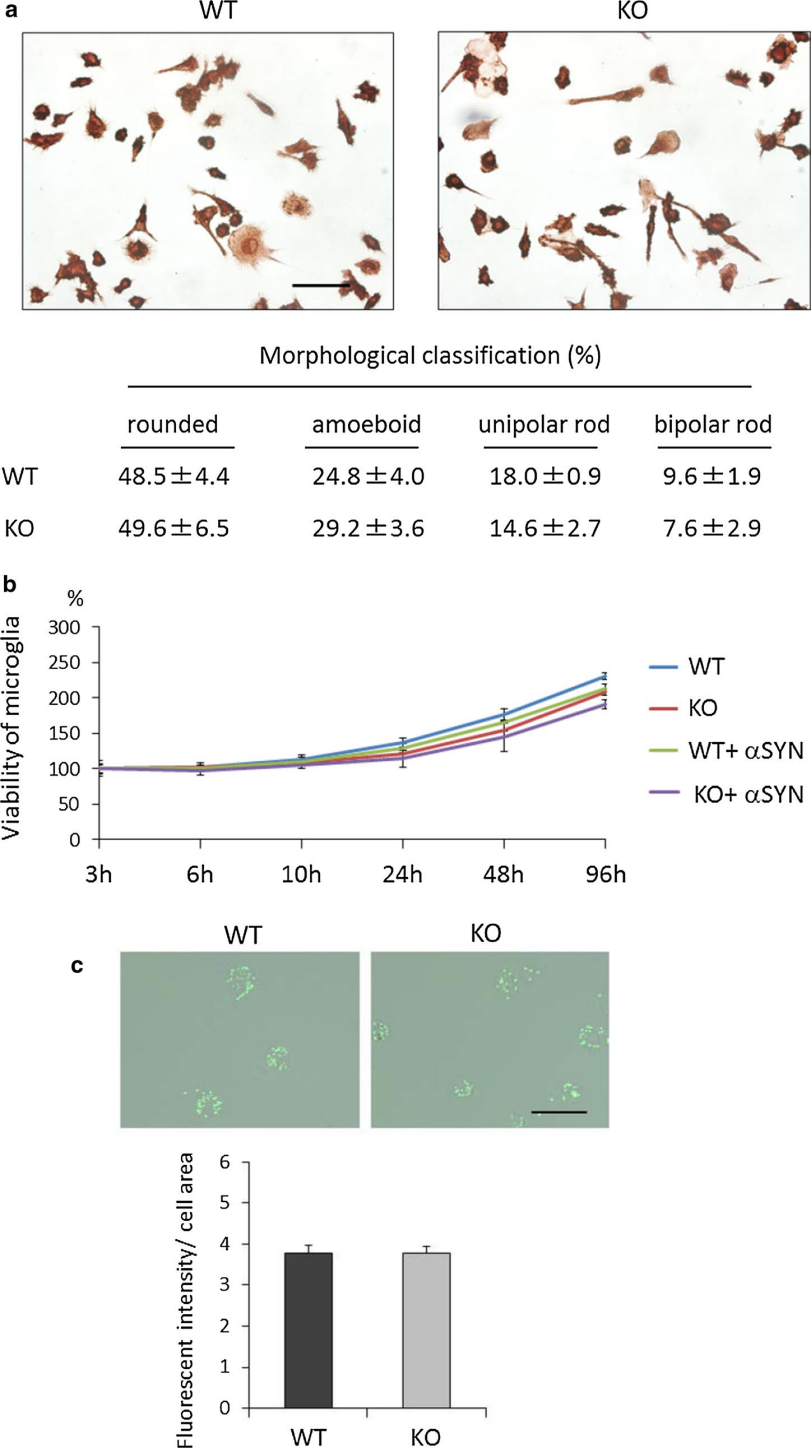
Characterization of LRRK2-KO microglia. **a** Tomato lectin staining of LRRK2-KO and WT microglia. One hundred cells were counted per culture well. n = 3 culture wells per group. *Scale bar* 50 μm. **b** MTT assay for evaluating the viability of LRRK2-KO and WT microglia in the absence or presence of αSYN. The data represent percentage viability relative to culture day 0, and were assessed by ANOVA at each time point (3 h: F = 0.26, p = 0.852; 6 h: F = 1.26, p = 0.351; 10 h: F = 0.94, p = 0.465; 24 h: F = 3.61, p = 0.065; 48 h: F = 2.55, p = 0.129; and 96 h: F = 3.46, p = 0.071). n = 3 culture wells per group. **c** Phagocytosis assay using FITC-microbeads. *Upper panel* shows an image of microbead phagocytosis. *Scale bar* 50 μm. *Lower panel* shows the fluorescence intensity of microbeads taken up by microglia. Forty cells were analyzed per group. The data are expressed as mean ± SD and were assessed by Student’s *t* test. These experiments were carried out three times using independent primary microglia isolated from different mice, and a representative image and data are shown

**Fig. 2:**
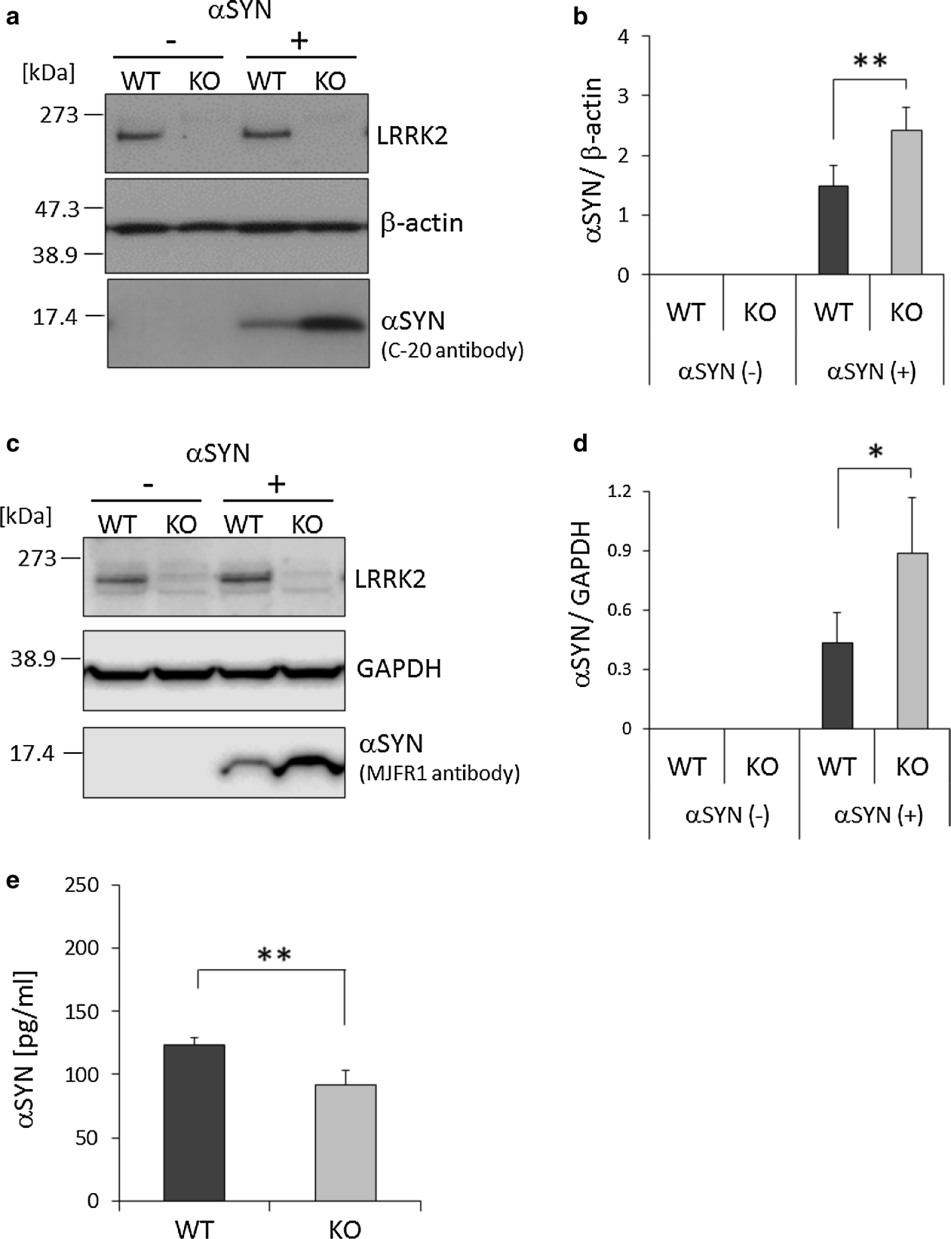
Increased αSYN uptake and degradation by LRRK2 KO microglia. **a** Western analysis of LRRK2-KO microglia treated with αSYN. Lysates were prepared from LRRK2- KO and WT microglia treated with or without αSYN and subjected to Western analysis using anti-LRRK2, β-actin, and αSYN (C-20) antibodies. **b** Quantified density of αSYN bands in (**a**) normalized by the density of β-actin. n = 4 culture wells per group. **c** Western analysis using anti-LRRK2, GAPDH, and αSYN (MJFR1) antibodies. **d** Quantified density of αSYN bands in (**c**) normalized by the density of GAPDH. n = 4 culture wells per group. **e** Residual αSYN in the culture media of WT and KO microglia. n = 8 culture wells per group. The amounts of αSYN were measured by ELISA 48 h after αSYN treatment. In all graphical representations, data are expressed as mean ± SD and were assessed by Student’s *t* test (KO vs. WT); *p < 0.05, **p < 0.01. All experiments were carried out three times using primary microglia isolated from independent mice, and a representative image and data are shown

To investigate the function of microglial LRRK2, primary microglia were prepared from LRRK2-KO mice and littermate WT control mice by Ni’s method [49]. Immunostaining with antibodies against Iba-1 (a microglial marker) and GFAP (an astroglial marker) revealed that the purity of the microglia was about 92%. No morphological differences in tomato lectin staining were observed between KO and WT microglia (Fig. 1a), and the MTT assay demonstrated no difference in viability between KO and WT microglia (Fig. 1b). Furthermore, the phagocytotic activities of KO and WT microglia did not differ from each other when analyzed using fluorescent latex beads (Fig. 1c). These results indicated that LRRK2 is not related to these microglial phenotypes. For evaluation of αSYN clearance, we performed αSYN uptake and clearance assay using recombinant αSYN. The αSYN treatment did not alter the protein level of LRRK2 (250 kDa) in WT primary microglia (Fig. 2a). Importantly, αSYN bands were detected only in the groups that had been treated with recombinant αSYN, indicating that the detected bands were not endogenous but exogenous recombinant αSYN. It was noteworthy that more αSYN was taken up by KO microglia than by WT microglia (Fig. 2b). The results were confirmed using different anti-αSYN and anti-GAPDH antibodies (Fig. 2c, d). Measurement of αSYN clearance showed that the level of residual αSYN in the culture medium of KO microglia was decreased more prominently than that in the medium of WT microglia (Fig. 2e). These results indicated that αSYN clearance by LRRK2-KO microglia is higher than that by WT microglia, suggesting that LRRK2 is a negative regulator of αSYN clearance in microglia.

### LRRK2 regulates endocytotic αSYN uptake

**Fig. 3:**
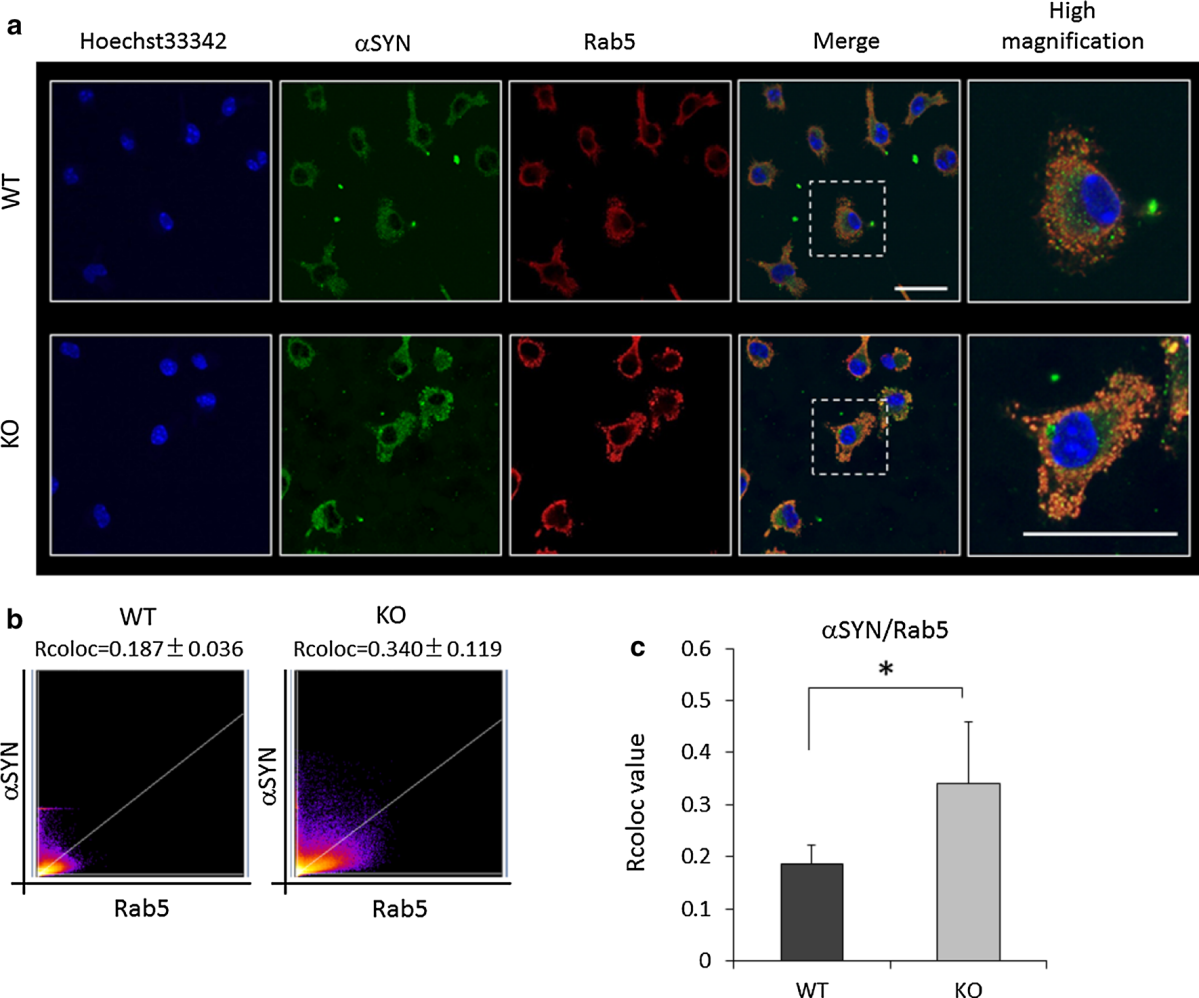
Increment of endocytotic αSYN uptake by LRRK2-KO microglia. **a** Confocal immunostaining images of αSYN and Rab5 in KO and WT microglia. *Scale bar* 50 μm. Higher-magnification views of the *boxed area* show the detail of immunopositive particles. *Scale bar* 20 μm. **b** Cytofluorograms for αSYN and Rab5 immunostaining evaluated using the high magnification views of KO and WT microglia. **c** The αSYN/Rab5 correlation coefficient (Rcoloc) values for KO and WT microglia. Ten cells were randomly chosen per group. In all graphical representations, data are expressed as mean ± SD and were assessed by Student’s *t* test; *p < 0.05. The experiment was carried out three times using primary microglia isolated from independent mice, and a representative image and data are shown

It has been reported that αSYN is internalized through the endocytotic pathway [50]. On the other hand, LRRK2 is associated with Rab5, a member of the Ras superfamily of small Rab GTPases and a component of the early endosome [51, 52]. To elucidate whether LRRK2 regulates endocytotic αSYN clearance through early endosomes, WT and KO microglia treated with recombinant αSYN were subjected to immunostaining with anti-αSYN and anti-Rab5 antibodies. Internalized αSYN was visualized as small particles spread widely in the all cell, and in keeping with the results of Western analysis, KO microglia showed higher amount of αSYN than WT microglia (Fig. 3a). Immunostaining for Rab5 demonstrated a pattern similar to that of αSYN. Interestingly, Rab5-positive early endosomes were obviously increased in KO microglia relative to WT microglia. Furthermore, as reported previously [53], some of the αSYN taken up was co-localized with Rab5-positive early endosomes. Relative assessment of co-localization between αSYN and Rab5 revealed that the Rcoloc value for KO microglia was higher than that for WT microglia (Fig. 3b, c). These results suggested that the increased uptake of αSYN by LRRK2-KO microglia is associated with an increase of early endosomes, i.e. LRRK2 is a down-regulator of the endocytotic pathway.

### Early endosomes are selectively increased in LRRK2-KO microglia

**Fig. 4:**
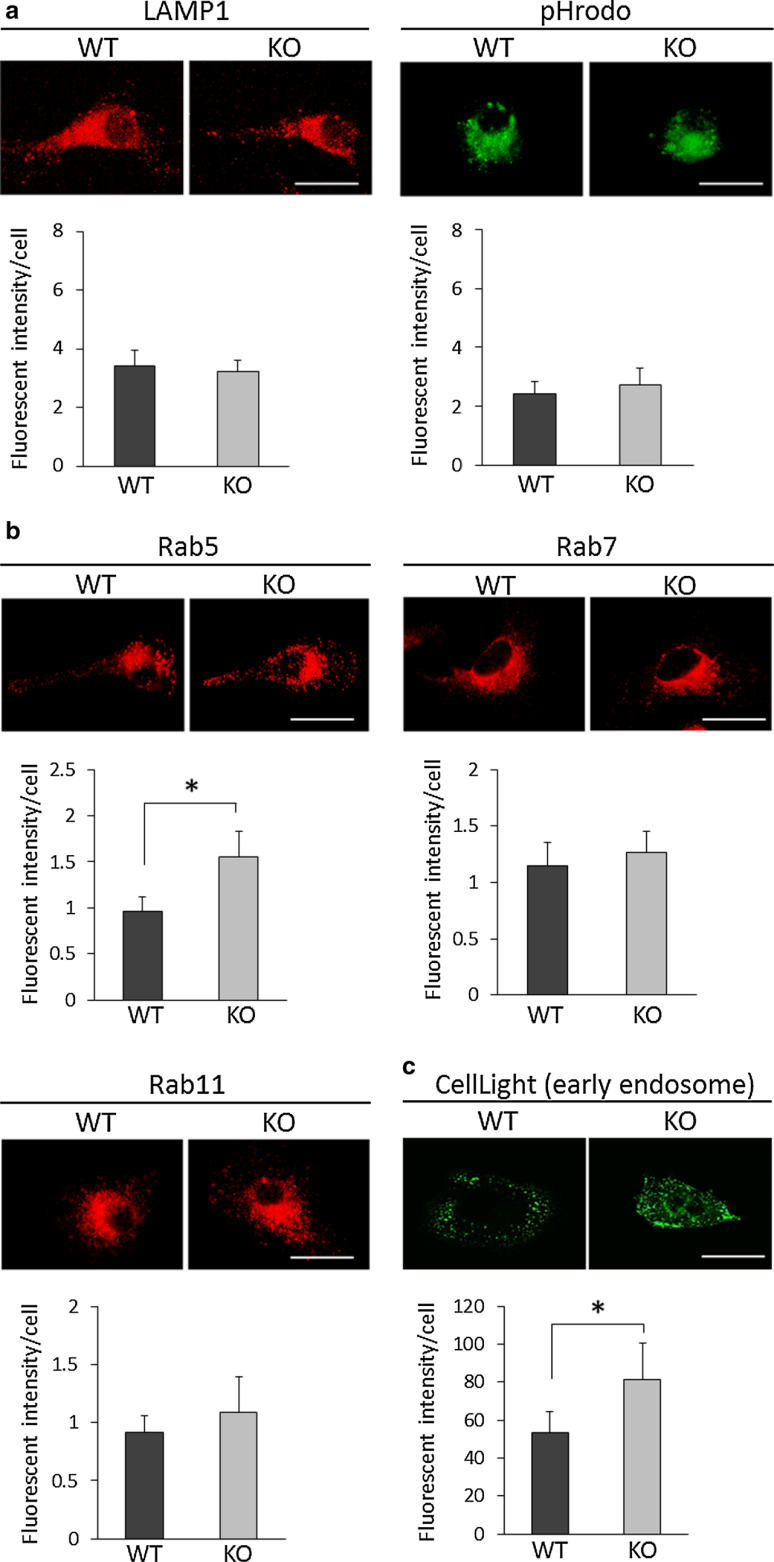
Increase of early endosomes, but not lysosomes, late, or recycling endosomes, in LRRK2-KO microglia. **a** Immunostaining for lysosomal markers in KO and WT microglia. Lysosomes and acidic lysosomes were visualized using anti-LAMP1 and pHrodo, respectively. **b** Immunostaining for endosomal markers in KO and WT microglia. Early endosomes, late endosomes, and recycling endosomes were visualized using antibodies against Rab5, Rab7 and Rab11, respectively. *Scale bar* 20 μm. Quantification of fluorescence intensity per cell showing an increase of Rab5-positive endosomes, but not Rab7- and Rab11-positive particles, in KO microglia. Twenty-five cells were analyzed per group. **c** Visualization of early endosomes using CellLight. Twenty-five cells were analyzed per group. In the graphical representation, data are expressed as mean ± SD and were assessed by Student’s *t* test; *p < 0.05. All experiments were carried out three times using primary microglia isolated from independent mice, and a representative image and data are shown

To investigate whether there is any abnormality in other components of the endosomal-lysosomal pathway in KO microglia, lysosomes were analyzed by immunostaining with anti-LAMP1 antibody (a lysosome marker) and a pH-sensitive molecular probe (a functional lysosome marker). In both analyses, the fluorescence intensities did not differ from that of WT microglia (Fig. 4a), indicating that the structure and function of the lysosome system were not altered in LRRK2-KO microglia. Immunostaining of the early endosome, late endosome, and recycling endosome using antibodies against the marker molecules Rab5, 7, and 11, respectively, revealed that the fluorescence intensity of Rab5-positive endosomes, but not that of Rab7- or 11-positive endosomes, was significantly increased in KO microglia relative to WT microglia (Fig. 4b). The increment of early endosomes in KO microglia was also confirmed by using CellLight (Fig. 4c).

**Fig. 5:**
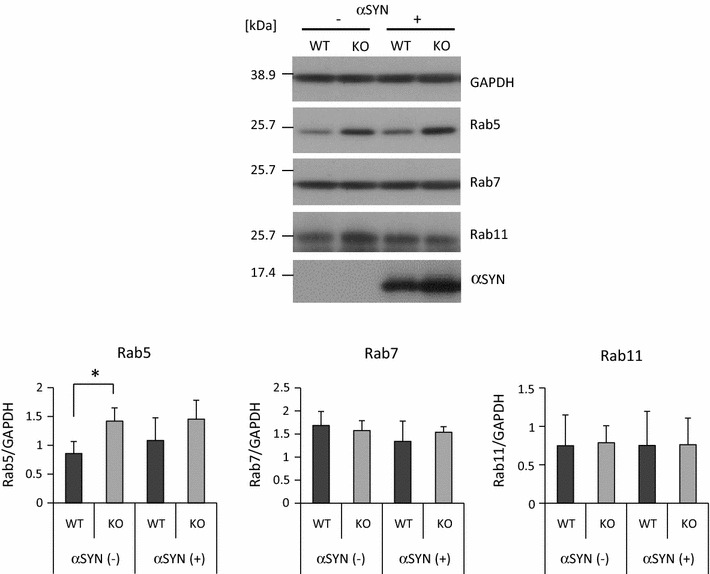
Higher Rab5 protein level in KO microglia than in WT microglia. Western analysis was performed to determine the protein levels of Rab5, Rab7 and Rab11 in KO and WT microglia treated with or without αSYN. The quantified density of each Rab was normalized by the density of GAPDH. n = 4 culture wells per group. In all graphical representations, data are expressed as mean ± SD and were assessed by ANOVA (KO vs WT). Rab5: F = 4.28, p = 0.028; Rab7: F = 0.96, p = 0.443; and Rab11: F = 0.01, p = 0.999. The experiment was carried out three times using primary microglia isolated from independent mice, and a representative image and data are shown

Western analysis using the same antibodies confirmed that the level of Rab5 protein was higher in KO microglia than in WT microglia (Fig. 5). After treatment with recombinant αSYN, an increase in the level of Rab5 protein in KO microglia, although not statistically significant, was also observed. These results indicated that early endosomes, but not other endosomes or lysosomes, are increased in LRRK2-KO microglia.

### LRRK2 controls the primary endocytotic process in microglia

**Fig. 6:**
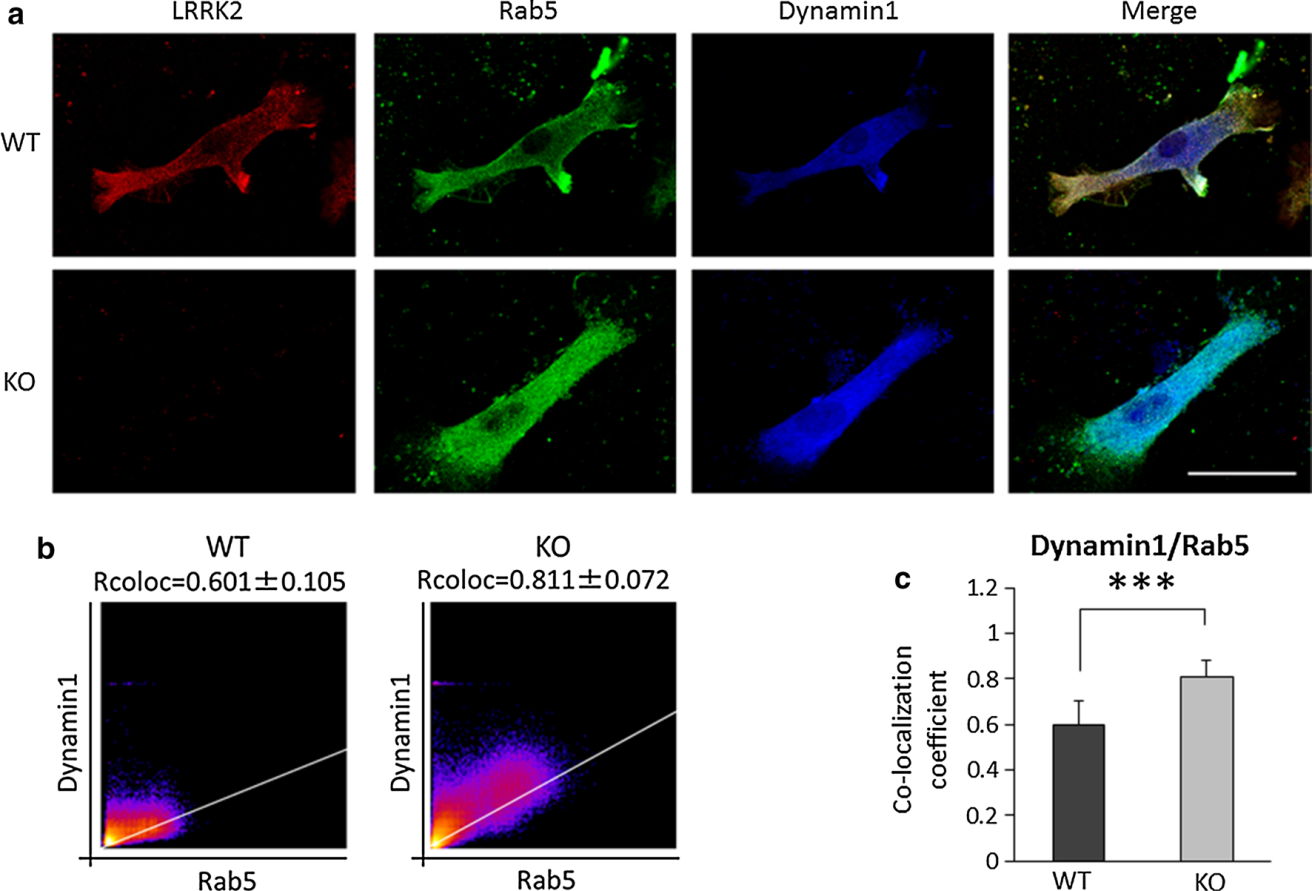
Increased co-localization between Rab5 and dynamin 1 in LRRK2-KO microglia. **a** Comparison of co-localization of Rab5 and dynamin 1 between KO and WT microglia. Confocal immunostaining images show LRRK2-, Rab5-, and dynamin1-immunopositive particles in the KO and WT microglia. *Scale bar* 10 μm. **b** Cytofluorograms of dynamin 1 and Rab5 immunostaining in KO and WT microglia. **c** The Rcoloc values of dynamin1/Rab5 in KO and WT microglia. Ten cells were analyzed per group. In the graphical representation, data are expressed as mean ± SD and were assessed by Student’s *t* test; ***p < 0.005. All experiments were carried out three times using primary microglia isolated from independent mice, and a representative image and data are shown

Recently, LRRK2 has been reported to associate with dynamin 1 in SH-SY5Y neuronal cells [54]. Dynamin 1 is one of the dynamin GTPase superfamily of molecules that modulate fission of the cytoplasmic membrane in the endocytosis pathway. To investigate how LRRK2 knockout affects dynamin 1, triple immunofluorescence staining with anti-LRRK2, anti-Rab5, and anti-dynamin1 antibodies was performed on KO and WT microglia. All staining was visualized as particles, and co-localization among LRRK2, Rab5, and dynamin1 was observed in primary microglia (Fig. 6a), in accordance with the previous study conducted using SH-SY5Y cells. Analysis of relative staining intensity showed that the Rcoloc value for Rab5 and dynamin 1 was increased in LRRK2-KO microglia relative to WT microglia (Fig. 6b, c). These results indicated that LRRK2 down-regulates the coordination between Rab5 and dynamin 1, resulting in reduced production of early endosomes.

## Discussion

In the present study, we clarified for the first time the role of LRRK2 in the process of αSYN clearance in microglia. Primary microglia isolated from LRRK2-KO mice exhibited increased uptake and clearance of αSYN and an increased immunostaining of Rab5-positive early endosomes, but not other endosomes or lysosomes. KO microglia also showed an increased degree of co-localization between Rab5 and dynamin 1. This study provides the first evidence that LRRK2 in microglia has a negative regulatory role in αSYN clearance by the early endocytosis pathway.

Because LRRK2-KO microglia showed up-regulation of the endocytosis pathway in both the presence and absence of recombinant αSYN, i.e., independently of exogenous αSYN, it is likely that autonomous up-regulation of endocytosis in LRRK2-KO microglia is one of the mechanisms for the increment of αSYN uptake and clearance. A recent study has reported that Rab5b, an isoform of Rab5, is phosphorylated by LRRK2 at Thr 6, resulting in a decreased endocytosis rate in Hela cells [55]. It is possible that up-regulation of the endocytosis rate in KO microglia could be due to the lack of phosphorylation of Rab5b. Our findings also suggested that enhancement of the coordination between Rab5 and dynamin 1 is another mechanism for the up-regulation of Rab5-positive early endosomes. It has been shown that G2019S-mutant LRRK2 with higher kinase activity inhibits the co-localization between Rab5 and dynamin 1 [54]. Given that the kinase activity of LRRK2 down-regulates the co-localization between Rab5 and dynamin 1, knockout of LRRK2 may enhance their co-localization and lead to an increase of Rab5-positive early endosomes. As an another possible mechanism, LRRK2 may regulate the amount of the Rab5 molecule itself. It has been reported that macrophages of the LRRK2-KO mouse exhibit an increase of IL-6 production through increased nuclear translocation of NFAT [43]. Because IL-6 is known to increase Rab5, but not other Rabs [56], it is possible that the elevated level of IL-6 in KO microglia may selectively increase Rab5-positive early endosomes and consequently promote the coordination between Rab5 and dynamin 1.

In contrast to our study, it has recently been reported that the protein level of αSYN was comparable between LRRK2-KO and WT rats that overexpress αSYN after viral transfection [48]. It is possible that the amount of virally overexpressed αSYN might far exceed the capacity of microglia to clear it. LRRK2 KO has also been reported to abrogate the neurodegeneration induced by αSYN overexpression in this strain of rat. It is possible that a decreased inflammatory response resulting from lack of LRRK2, rather than up-regulated αSYN clearance, might be responsible for this alleviation of neurodegeneration.

It has been shown that an excess amount of αSYN induces neuronal death [11–13], and that continuity of this process will eventually cause PD [57]. Because αSYN is known to be transmitted from cell to cell, and microglia have the highest ability to clear it [16–18, 29], αSYN clearance by microglia, in addition to that by neurons themselves, would be of crucial importance for maintaining a healthy brain environment.

## Conclusions

We have demonstrated that LRRK2 in microglia may function as the offending molecule responsible for neurodegeneration, in terms of down-regulation of αSYN clearance. In other words, inhibition of LRRK2 would be a potential therapeutic approach for PD.

## Methods

### Animals

LRRK2 exon 41-KO mice, developed by Hinkle and co-workers [58], on C57BL/6 J and littermate wild-type (WT) mice were used. The mice were housed in a light- and temperature-controlled room with water and food available ad libitum. For sacrifice, mice were euthanatized by cervical dislocation or exsanguination. All procedures had been approved by the Animal Experimentation and Ethics Committee of Kitasato University.

### Antibodies

The following primary antibodies were used: rabbit polyclonal anti-α-synuclein antibody (C-20, Santa Cruz Biotechnology), rabbit monoclonal anti-α-synuclein antibody (MJFR1, Abcam), mouse monoclonal anti-α-synuclein antibody (4D6, Abcam), rabbit monoclonal anti-LRRK2 antibody (MJFF2, Epitomics), rabbit polyclonal anti-Iba-1 antibody (Wako), horseradish peroxidase (HRP)-labeled mouse monoclonal anti-β-actin antibody (ab20272, Abcam), rabbit monoclonal anti-glyceraldehyde-3-phosphate dehydrogenase antibody (GAPDH; 14C10, Cell Signaling Technology), mouse monoclonal glial fibrillary acidic protein (GFAP) antibody (2A5, Abcam), mouse monoclonal anti-Rab5 antibody (Rab5-65, Abcam), rabbit monoclonal anti-Rab5 antibody (C8B1, Cell Signaling Technology), rabbit monoclonal anti-Rab7 antibody (D95F2, Cell Signaling Technology), rabbit monoclonal anti-Rab11 antibody (D4F5, Cell Signaling Technology), and mouse monoclonal anti-LAMP1 antibody (H4A3, Abcam).

### Isolation of primary microglia

Primary microglia isolation was performed according to the method of Ni [49]. Briefly, neonatal brains were obtained from WT and LRRK2-KO mice at 2 days of birth, then cortex sections were dissected out under a microscope and digested with papain for 20 min at 37 °C. The dispersed cells were suspended in growth medium (DMEM; Sigma, 10% FCS; Gibco, penicillin/streptomycin) and cultured at 40,000 viable cells/cm^2^. After mixed glial cells had become 100% confluent (about 10 days after plating), the culture flask was tapped gently until most of the microglia became detached. The isolated microglia were then cultured in growth medium at 10,000 viable cells/cm^2^ on poly-l-lysine (Sigma)-coated cover slips or in 24-well plastic tissue culture plates. The cells were used for experiments two days after plating. The purity of the microglia was about 92% when determined by immunostaining with the anti-Iba-1 antibody and anti-GFAP antibody.

### Measurement of αSYN uptake

Primary microglia were treated with 5 μg/ml recombinant αSYN [γ-peptide] for 24 h. Uptake of recombinant αSYN was analyzed by Western analysis and immunofluorescence staining using the anti-αSYN antibody.

### Measurement of αSYN clearance

Primary microglia were treated with 250 pg/ml recombinant αSYN for 48 h. After incubation, the level of residual αSYN in the culture medium was measured with a SensoLyte™ Anti-alpha-Synuclein Quantitative ELISA Kit (Anaspec) in accordance with the manufacturer’s protocol.

### Western blotting

Microglia were lysed in TNE buffer [10 mM Tris–HCl buffer (pH 7.6) containing 150 mM NaCl, 1% Nonidet P-40, 1 mM EDTA, 1 mM phenylmethylsulfonyl fluoride, and protease inhibitor cocktail (Roche)] and incubated at 4 °C for 1 h. The cell lysate was obtained by centrifugation at 13,000 rpm for 15 min at 4 °C, then subjected to sodium dodecyl sulfate polyacrylamide gel electrophoresis (SDS-PAGE) using 5–20% gradient e-PAGEL (ATTO), and blotted onto polyvinylidene fluoride (PVDF) membranes. The membranes were blocked in 2% skim milk in phosphate-buffered saline (PBS)—0.1% Tween 20 for 60 min at room temperature, and then probed with the appropriate primary antibodies for 24 h at 4 °C. After incubation with secondary antibodies for 30 min at room temperature, protein bands were visualized using an ECL Western Blotting Detection Kit (GE Healthcare). The intensity of the protein bands was analyzed using NIH ImageJ software (http://rsbweb.nih.gov/ij/).

### Tomato lectin staining

Primary microglia attached to cover slips were fixed with 4% paraformaldehyde for 30 min at room temperature and reacted with biotin-conjugated tomato lectin (Vector Laboratories) for 30 min at room temperature. Avidin/biotinylated HRP complex and ImmPACT DAB Substrate (Vector Laboratories) were used for detection.

### Immunofluorescence staining

After treatment with 2% BSA in PBS—0.2% Triton X-100 for 60 min at room temperature to block non-specific binding, the fixed microglia were incubated with primary antibodies for 24 h at 4 °C, and subsequently with appropriate secondary antibodies for 30 min at room temperature. Nuclear staining was performed with 2′-(4-ethoxyphenyl)-5-(4-methyl-1-piperazinyl)-2,5′-bi-*1H*-benzimidazole (Hoechst 33342, Dojindo). Visualization of early endosomes using CellLight Early Endosomes-GFP (Molecular probes) was conducted according to the manufacturer’s protocol. Fluorescent signals were observed using a Nikon C2 Si confocal microscope system using 525–575, 595–635, and 445–475 nm band pass filter to obtain the signal by 488 nm-, 561 nm-, and UV-laser excitation, respectively. Pearson’s correlation coefficient (Rcoloc) value was given by Coloc 2-plugin in ImageJ for each cell.

### 3-(4,5-Dimethylthiazol-2-yl) 2,5-diphenyltetrazolium bromide (MTT) assay

The culture supernatants of primary microglia cultured for 1, 3, 6, 12, 24, and 48 h with or without recombinant αSYN were assayed for viability using a Cell Counting Kit-8™ (Dojindo) in accordance with the manufacturer’s protocol.

### Phagocytosis assay

Primary microglia were incubated with fluorescent latex beads (0.0025%, Sigma) for 1 h. The cells were washed with PBS, fixed with 4% paraformaldehyde, and mounted on glass slides using ProLong^®^ Gold antifade reagent (Molecular Probes). Fluorescence intensity per cell was determined using ImageJ software.

### Statistical analysis

All data are expressed as mean ± S.D. Significance of differences was assessed by Student’s *t* test for two comparisons and one-way ANOVA for multiple comparisons.

## Abbreviations

αSYN: α-synuclein
GAPDH: glyceraldehyde 3-phosphate dehydrogenase
GFAP: monoclonal glial fibrillary acidic protein
KO: knockout
LRRK2: leucine-rich repeat kinase 2
MTT assay: 3-[4,5-dimethylthiazol-2-yl]-2,5 diphenyl tetrazolium bromide assay
PBS: phosphate-buffered saline
PD: Parkinson’s disease
PVDF: polyvinylidene fluoride
SDS-PAGE: sodium dodecyl sulfate polyacrylamide gel electrophoresis
TLR: toll-like receptor
WT: wild-type
